# Pictorial Essay of Cervical Duplex Ultrasonography

**DOI:** 10.24908/pocus.v7i2.15635

**Published:** 2022-11-21

**Authors:** Siddharth Bhattacharjee, Richa D Jain, Lokesh Bathala, Anuradha HK, Vijay K Sharma

**Affiliations:** 1 Dept. of Neurology, Aster CMI Bangalore; 2 Dept. of Radiology, Aster CMI Bangalore; 3 Division of Neurology, Yong Loo Lin School of Medicine, National University of Singapore, National University Hospital Singapore

**Keywords:** carotid duplex ultrasonography, Carotid Doppler in stroke, Carotid Doppler in dissection

## Abstract

**Objectives**: Cervical duplex ultrasonography (CDU) is a simple, non-invasive, portable technique, that provides valuable high-quality visual information about the integrity of the carotid and vertebral vessels, plaque morphology and flow hemodynamics. CDU is useful in the assessment and follow up of patients with cerebrovascular disease as well as other conditions like inflammatory vasculitis, carotid artery dissection and carotid body tumours. CDU is inexpensive and invaluable in smaller centres. **Methods**: CDU was performed in all patients in both longitudinal and transverse planes in the out-patient clinic. Brightness mode (B-mode) and Doppler waveforms were obtained. Relevant findings were presented. **Results**: CDU provides real time visualisation of plaque characteristics and follow up, hemodynamic characteristics in Takayasu arteritis, visualisation of dissection. **Conclusion**: With availability of MR/CT angiography, CDU can be an adjuvant in follow up, triage and early bed-side diagnosis of the vascular diseases. We present our experience with CDU in the out-patient clinics in this pictorial essay.

## Introduction

Carotid arteries are the major vessels supplying blood to the brain. Early identification of a diseased carotid artery is essential for preventing life threatening morbidities such as stroke. CDU is a non-invasive, out-patient technique to assess the structure of the carotid wall, intima media thickness as well as flow velocity of the common carotids, internal carotids, and the vertebral arteries 1. In its initial days, carotid duplex ultrasonography replaced oculopneumoplethysmography (OPG) as the only non-invasive means for measuring pressure drop across a carotid lesion. CDU used the Bernoulli principle to describe pressure gradients across localised narrowing in terms of square of the flow velocity  2. It was later established that the use of peak systolic and end diastolic velocities, and ICA/CCA peak systolic velocity ratio was more accurate in estimating the level of carotid stenosis. Further advancements in ultrasound equipment over the last few decades with spatio-temporal filtering to reduce image noise, auto-segmentation of images with integrated Doppler imaging provides high quality visualisation of carotid wall integrity, flow characteristics and real time plaque morphology 3. While digital subtraction imaging remains the standard for estimating degree of stenosis for interventional procedures like carotid endarterectomy and stenting, CDU remains the first line non-invasive imaging technique for not only estimating stroke risk for primary prevention of stroke, but also to assess response to therapy. Additionally, CDU helps in visualising carotid body tumour, carotid dissections, and flow characteristics in such conditions 4. More recently, 3-dimensional ultrasonography is being used to measure the carotid total plaque area and plaque volume accurately and for evaluating the effects of therapy on atherosclerotic carotid plaques 5. With the continuous integration of artificial intelligence and machine learning techniques into healthcare, the inevitable future is more enhanced automatic detection of plaque characteristics and point-of-care diagnosis of vascular diseases. Hybrid deep learning (HDL) paradigms for carotid plaque characterisation are currently undergoing multicentre trials and showing promising accuracy 6, 7. This will cut-down the need for operator expertise for standard interpretation of ultrasonography images, especially in work intense areas like the emergency intake of a hospital and in out-patient clinics without access to vascular sonologists.

We present a pictorial essay of CDU images in various out-patient situations, which helped in making an early diagnosis and appropriate management. 

## Technique and Methods

All patients were positioned in the supine position with the sonographer at the head-end of the bed. CDU was performed both in the transverse as well as longitudinal planes. The transverse plane enables identification of the jugular vein, Common carotid arteries (CCA), carotid bulb, carotid bifurcation, external and internal carotid arteries (ECA, ICA). CDU is started from the most proximal CCA and its course is followed to the carotid bifurcation and ICA is traced as distally. The ICA can be differentiated from the ECA by its more posterior position, the lower resistive pattern on doppler ultrasonography and the “temporal tapping” method. Once the ICA is visualised, longitudinal images are observed. Brightness mode (B-mode) imaging helps visualise intima media thickness and plaque morphology in terms of plaque echogenicity, plaque ulceration and mobility. These findings were then confirmed by colour mode. Finally, Doppler spectral waveforms were obtained to determine velocity 8. 

It is important to obtain multiple spectral analyses of each vessel, including the pre-stenotic and the post-stenotic area, in addition to the area of stenosis itself, to appreciate indirect hemodynamic effects of the stenosis  9. Intima media thickness on 2-dimensional grey scale imaging, of more than 1 mm was considered abnormal in the common carotid arteries 10. All CDU techniques were performed by trained neuro-sonologists with over 10 years of experience in the field, in Neurology out-patient clinic scenarios.

Verbal consent was obtained from every patient prior to examination and the code of ethics as per declaration of Helsinki were followed. Since no patient details/identity is being disclosed in our century, the need for institution ethics committee approval was waived. No IRB number was provided. 

## Case 1: Carotid stenosis

The internal carotid artery (ICA) stenosis is responsible for up to 20% of all strokes 11. Symptomatic hemodynamically significant stenosis in the ICA can cause cerebral ischemia due to thromboembolism or hemodynamic insufficiency. 

**Figure 1:**
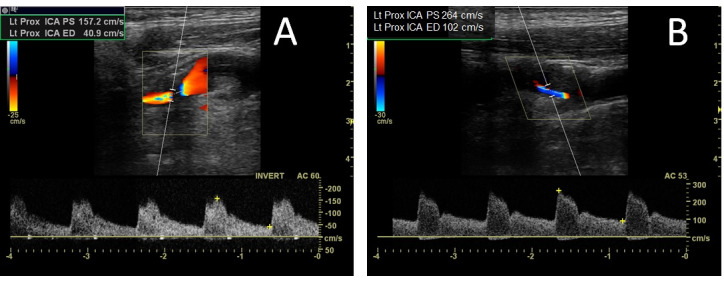
Carotid duplex ultrasonography findings from patients with carotid stenosis. A) Duplex findings obtained from a 68 year-old gentleman, who presented with transient right sided weakness. A heterogeneous plaque was noted at the carotid bulb with moderately elevated flow velocities (peak systolic velocity (PSV) of 157.2cm/s and end-diastolic velocity (EDV) of 40.9cm/s), consistent with 50-69% focal stenosis. Optimization of his diabetes and lipid control medications, along with short-term dual antiplatelet therapy prevented any further cerebral ischemic event. B) Duplex findings from a 57 year-old diabetic and hypertensive gentleman, who presented with a right sided subcortical ischemic stroke. A large homogeneous plaque was noted in right proximal ICA with focally elevated flow velocities (PSV 204cm/s and EDV 102cm/s), consistent with >70% stenosis. He underwent uneventful early carotid endarterectomy and has remained well during the last 2 years on follow up.

Duplex imaging is an established tool for grading the carotid artery stenosis and considered as the primary diagnostic modality. Duplex criteria for quantifying carotid artery stenosis have been developed primarily through comparisons of duplex derived spectral waveforms with contrast arteriogram. The sensitivity and specificity of duplex ultrasonography for the diagnosis of carotid stenosis is between 90 to 95% 12. Currently, detection of specific threshold levels of the ICA stenosis appears to be most clinically important. Consensus criteria recommended by the Society of Radiologists in Ultrasound (SRU) are most frequently used to grade the severity of ICA stenosis 13. Duplex findings from patients with 50-69% stenosis and >70% stenosis are presented in Figure 1.

Carotid plaques underlying the stenotic lesion play an important role in contributing towards the ischemic cerebral events. Various morphological characteristics of the carotid plaques have been described as determinants of their ‘embolic potential’. In addition to the size of the plaque, these include plaque composition, the type of the fibrous cap overlying the plaque, intraplaque hemorrhage and ulceration on the surface 14, 15.

**Figure 2:**
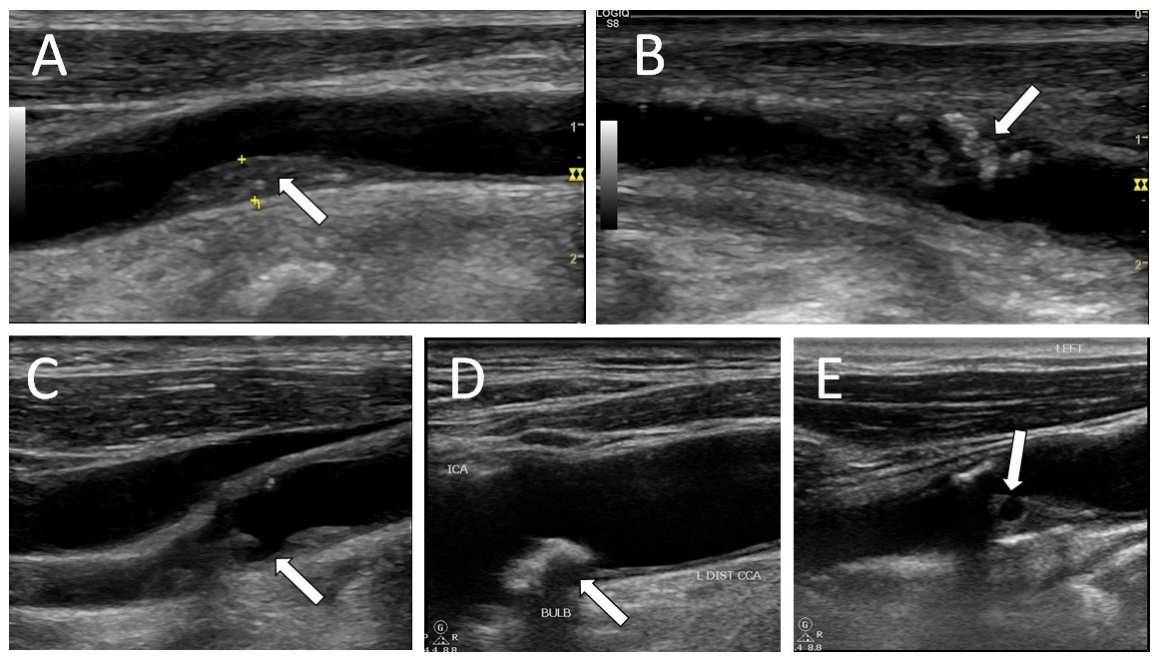
B-mode ultrasound appearance of various types of carotid plaques. A) a largely homogeneous plaque with a thin fibrous cap. B) a heterogeneous plaque with patchy areas of calcification. C) a large soft plaque with surface ulceration and ulcer crater, obtained from a patient with recurrent transient ischemic attacks. D) a calcified plaque with shadowing. Such plaques are considered to be stable and have a good prognosis. E) a large homogeneous plaque with an area of intraplaque haemorrhage, often associated with high risk of cerebral ischemic events.

It is well established in literature that echolucent plaques on grey scale imaging have an increased risk of ischemic stroke 5, 16, 17. Contrast enhanced ultrasonography (CEUS) and use of superb microvascular imaging ultrasound without contrast, can also be used to detect carotid plaque neovascularisation. CEUS is also superior in demonstrating plaque ulceration 18. Ultrasound appearances of various types of plaques are presented in Figure 2.

## Case 2: Carotid body tumour (CBT)

Paragangliomas are rare vascular tumours derived from the paraganglia tissues originating from the neural crest, which include sympathetic and parasympathetic paraganglia. Most of the sympathetic paragangliomas arise within the adrenal glands and abdomen while parasympathetic paragangliomas are predominantly found in head and neck regions. Carotid body tumours are neuroendocrine neoplasms which arise near the carotid bifurcation within glomus cells derived from the embryonic neural crest. They represent 60-70% of paragangliomas of the head and neck ^19, 20, 21^.

**Figure 3:**
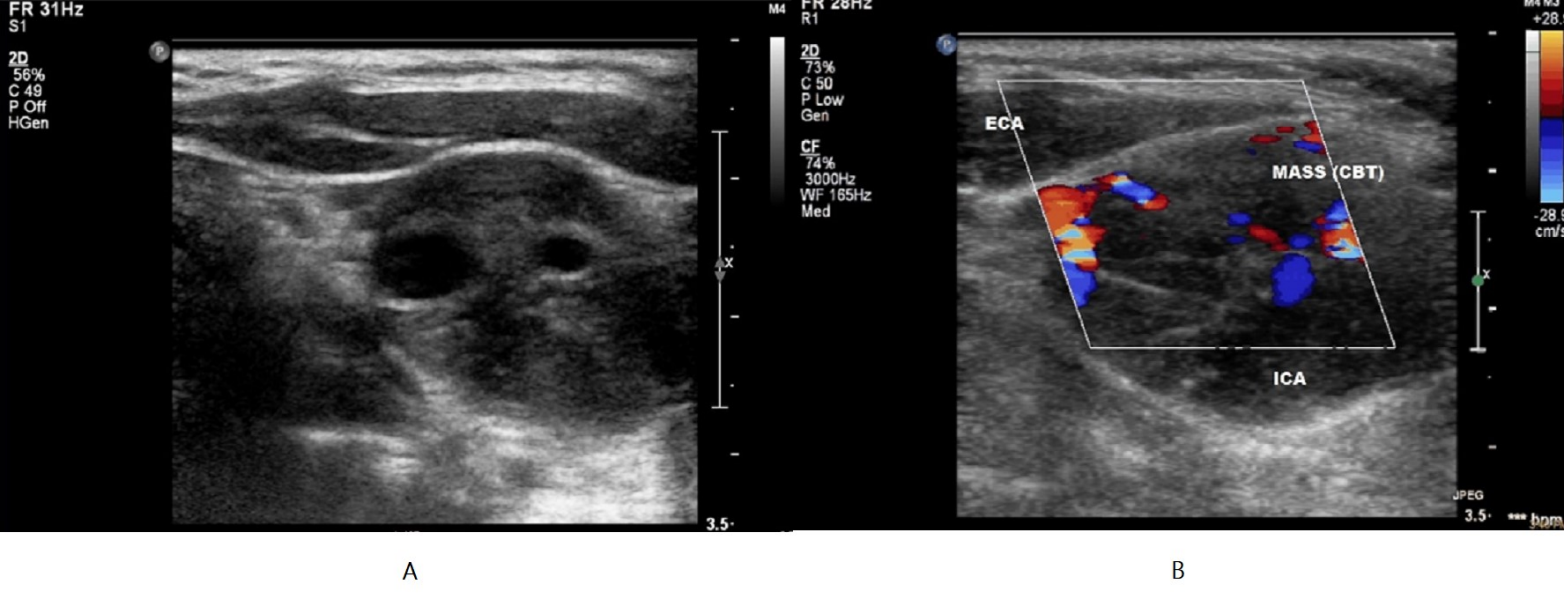
34 year-old lady with a right sided neck mass. A) and B) Carotid ultrasound showing a hypoechoic heterogeneous mass at the carotid bifurcation with internal vascularity.

**Figure 4:**
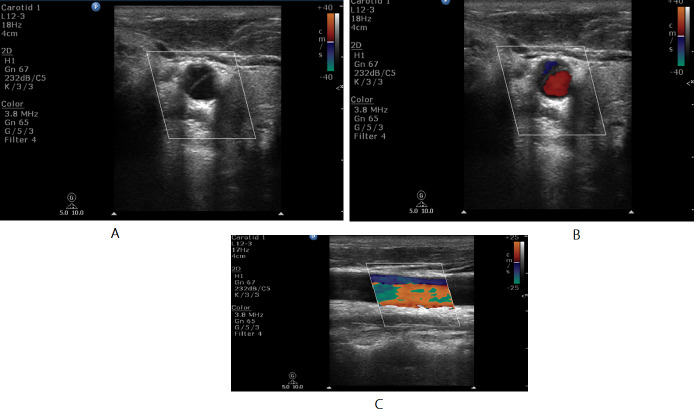
A 41 year-old lady with left sided neck pain. A) and B) CDU axial section through the CCA shows a linear dissection flap with ‘yin and yang’ flow pattern. C) Longitudinal view CDU of dissection with colour flow Doppler.

We present a 34 year-old lady with a painless pulsatile mass on the right side of neck. CDU revealed a characteristic vascular solid hypoechoic mass in the carotid bifurcation region (Figure 3A and 4B). It was a type II CBT (Shamblin classification) with splaying of carotid bifurcation. 

During CDU, vascularity and location are the two key factors used to differentiate carotid body tumours from other neck masses, such as lymphomas, metastatic tumours, thyroid lesions, submandibular salivary gland tumours, and branchial cleft cysts 22.

Shamblin classification includes Type I tumours, which are small lesions that do not splay the carotid bifurcation, Type II tumours are larger and significantly splay the carotid bifurcation, but do not circumferentially encase the carotid arteries. Tumours that are large, encapsulate the internal or external carotid arteries, and often adhere or incorporate the adjacent cranial nerves are Type IIIa, whereas Type IIIb tumours include tumours of any size that are intimately adherent to the carotid vessel 23, 24.

## Case 3: Carotid artery dissection

Dissections arise due to a tear in the intima layer of the vessel, which can be life threatening. We describe a 41 year-old lady, who presented with a short history of pain in the left side of neck and scalp. She had taken her dog for a walk and the pain started suddenly after a jerk to the neck when she had to suddenly pull the leash hard to stop the dog from running. 

Upon arrival, her clinical examination was normal. However, CDU revealed dissection in the left distal CCA.

CDU demonstrated a thin echogenic intimal flap dividing the vessel into true and false lumen (Figure 4). Due to the mass effect from the false lumen, Doppler spectra may demonstrate a high-resistance pattern, which may progress to bidirectional systolic flow with minimal diastolic flow 25.

## Case 4: Takayasu arteritis

Takayasu arteritis (TA) is a chronic granulomatous large vessel vasculitis that affects the aorta, its main branches, and pulmonary arteries. TA is characterized by the involvement of all arterial layers (i.e. pan-arteritis) with a variable inflammatory infiltrate 26. CDU in such cases help in characterising the inflammation of vessel wall as well as for monitoring the hemodynamic changes in response to treatment. Specifically, during active disease, CDU can detect characteristic long segments with homogenous, mid-echogenic, circumferential arterial wall thickening, known as the “Macaroni sign”. During inactive disease, as the circumferential wall thickening reduces, and hyperechogenic stripes lining the innermost wall layer are observed. This is different from the inhomogeneous, asymmetric, partly calcified arterial wall changes, which are typically observed in atherosclerosis  27. 

**Figure 5:**
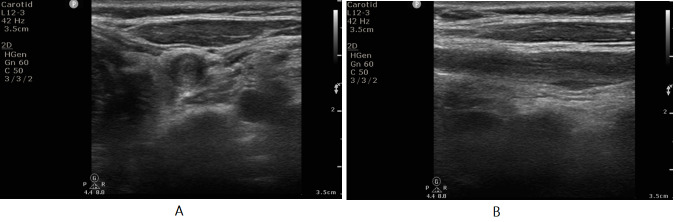
A 31 year-old gentleman, hypertensive, presented with multiple TIA like episodes. A) and B) Short axis and long axis views on CDU showing increased intimal thickness of left common carotid artery.

**Figure 6:**
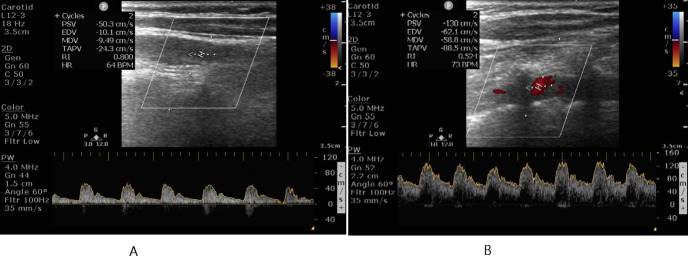
A 31 year-old gentleman, hypertensive, presented with multiple TIA like episodes. A) and B)CCA and ICA showing peak systolic velocity of 50 cm/s and 130 cm/s respectively. ICA/CCA PSV ratio more than 2.

We present a 31 year-old gentleman with background history of systemic hypertension who presented with multiple transient ischemic attacks (TIA). Evaluation revealed high ESR and differential systolic blood pressure of 14 mmHg. Carotid ultrasound revealed thickened left common carotid artery and elevated flow velocities in left internal carotid artery (Figure 5 and Figure 6).

**Figure 7:**
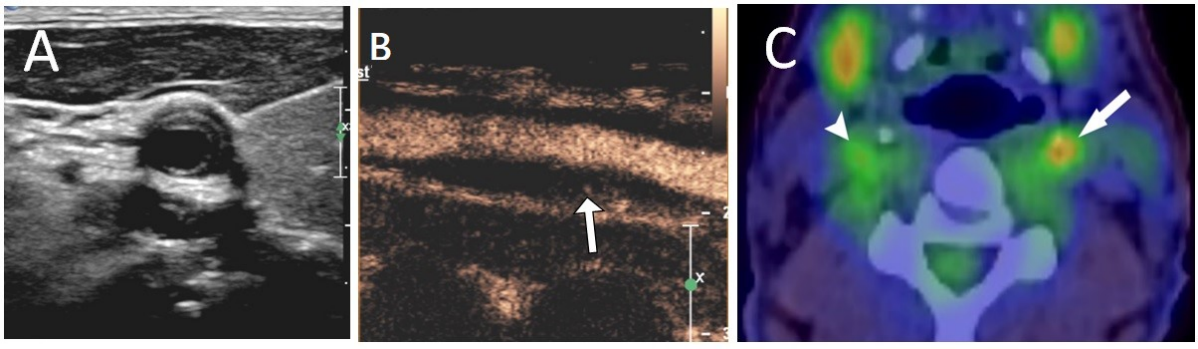
Comparison of imaging modalities showing carotid wall thickening. A) Short axis view showing thickening of carotid wall. B) Long axis view with contrast showing microbubbles of ultrasound contrast, suggestive of neovascularisation. C) PET scan confirming inflammatory process on the left carotid (arrow), compared to the normal right carotid (arrowhead).

Apart from high resolution vessel wall magnetic resonance imaging, ultrasonography is the only imaging modality that can aid in the assessment of carotid arterial wall. Thickening of carotid wall may be seen in arteritis other than Takayasu arteritis as well (Figure 7A). In patients with active inflammation of the carotid wall or an atheromatous plaque, carotid ultrasound may be performed with intravenous contrast agents. Appearance of microbubbles of ultrasound contrast agents in the arterial wall or under-surface of the plaque is abnormal and represents neo-vascularization (Figure 7B). Such inflammatory process can be confirmed with positron emission tomography (Figure 7C).

## Case 5: Subclavian steal syndrome

Subclavian-vertebral artery steal syndrome (SSS) is the hemodynamic phenomenon of blood flow reversal in the vertebral artery (VA) due to significant stenosis or occlusion of the proximal subclavian artery or the innominate artery that may result in significant vertebrobasilar ischaemia by siphoning blood from the vertebral artery 28, 29. Ultrasound of the carotids and vertebral artery can detect the various stages of subclavian steal: 

***1. Pre-steal***– the earliest stage in the disease process and manifests as transient mid-systolic flow deceleration or systolic notch, which are referred to as the ‘‘bunny waveform”. However, flow remains anterograde throughout the cardiac cycle. 

***2. Partial steal*** – exhibits flow reversal during systole

***3. Complete subclavian steal ***– exhibits retrograde flow in vertebral artery throughout the cardiac cycle 30.

**Figure 8:**
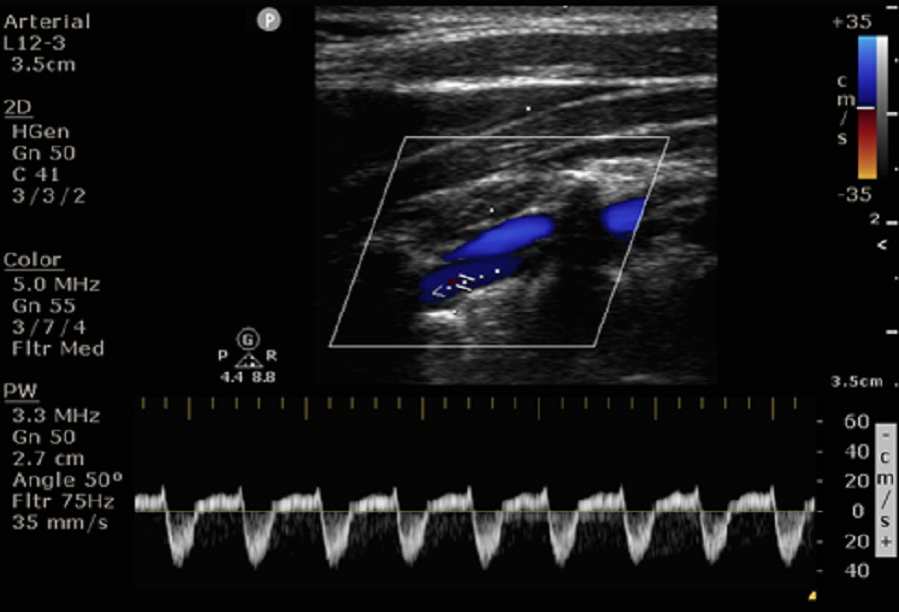
A 68 year-old gentleman presented with vertigo and recurrent syncope. Carotid Doppler of left vertebral artery showed flow reversal during systole as seen in partial steal.

**Figure 9:**
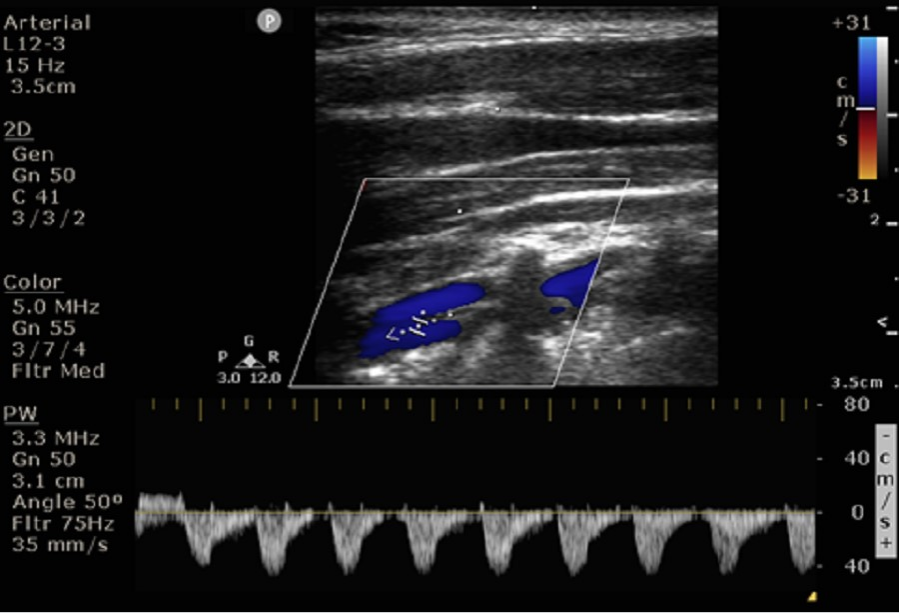
A 68 year-old gentleman presented with vertigo and recurrent syncope. Doppler ultrasound of left vertebral artery showing complete flow reversal during systole as seen in complete steal.

We present a 68 year-old gentleman who presented with vertigo and recurrent syncope. MRI of the brain with MR angiography and venography did not show any acute ischaemic changes. CDU exhibited intermittent complete flow reversal throughout the cardiac cycle (Figures 8, 9).

## Limitations

Though hybrid deep learning paradigms will reduce the need for operator expertise in the future, the technology is still years away from being available in standard ultrasonography devices. At present, operator dependence and image reproducibility are the main limitations of CDU. The imaging technique requires the operator to hold the probe at a constant 60°angle for CDU to avoid degradation of images due to “Doppler shift” 31. Further, CDU can over-estimate the degree of stenosis in patients with significant contralateral stenosis (>70%) due to increased compensatory flow 32. In addition, the specificity of CDU in patients with less than 70% stenosis (50-69%) is relatively low and such patients can benefit from opting for other imaging techniques like carotid angiography and DSA 33. Calcification, high bifurcation, or short neck anatomy causes limitation in evaluation of carotid bifurcation with CDU techniques. Additionally, it is not possible to evaluate distal ICA. 

In our experience, it takes about 2 years of CDU experience for a trained operator to be able to confidently report carotid ultrasonography images.

## Conclusion

We have presented our experience with carotid ultrasound in the out-patient clinics in this pictorial essay. Carotid ultrasound is a non-invasive, out-patient procedure that can be used for diagnosis and follow up of various vascular diseases. With availability of MR/CT angiography, CDU can be an adjuvant in follow up, triage and early bed-side diagnosis of the vascular diseases. CDU is inexpensive and invaluable in smaller centres. Though CDU has certain limitations, new technological advancements and use of artificial intelligence techniques promise to bridge the gap between expertise and routine interpretation of CDU images.

## Declaration of conflict of interests

The authors declare no potential conflicts of interest with respect to research, authorship, and/or publication of this article.

## Funding

The authors received no financial support for the research, authorship, and/or publication of this article.
